# Automated cementing quality detection using a domain-specific, multi-scale convolutional neural network

**DOI:** 10.1371/journal.pone.0337924

**Published:** 2025-12-09

**Authors:** Wenfa Yang, Shaoliang Sun, Yu He, Haoqiang Wu

**Affiliations:** 1 Anton Petroleum Technology (Group) Co., Ltd., Beijing, China; 2 CNPC Great Wall Drilling Company, Beijing, China; 3 Institute of Geology, No. 3 Engineering Technology Research Institute of Southwest Oil & Gas Field Company, PetroChina, Deyang, China; 4 China University of Geosciences, Wuhan, China; Khalifa University of Science and Technology, UNITED ARAB EMIRATES

## Abstract

Cementing quality is a key factor in ensuring the long-term safe production of oil and gas wells and preventing defects. Traditional cementing quality evaluation mainly relies on logging interpreters manually analyzing acoustic logging data, such as Variable Density Logging (VDL) images and acoustic amplitude curves. This process is highly dependent on personal experience, labor-intensive, and inefficient. To address these issues, this paper proposes an automated cementing quality detection method, CemQ-CNN, based on a Convolutional Neural Network (CNN). In this context, “intelligent” refers to the model’s ability to perform automatic classification from raw data, thereby increasing efficiency and consistency. This method constructs a multimodal input CNN model that can simultaneously process VDL images and acoustic logging curve data, achieving automatic, fast, and accurate classification of cementing quality. We collected and labeled 5,000 logging samples from 150 different wells across three distinct geological blocks, ensuring dataset diversity, categorizing them into three cementing quality levels: “good,” “medium,” and “poor.” By allocating 70% of the data for training, 15% for validation, and 15% for testing, our model demonstrated Good performance on the test set. Experimental results show that the proposed method achieves an overall classification accuracy of 95.7%, demonstrating robust performance across all three quality classes (‘Good’, ‘Medium’, and ‘Poor’), with a macro-average recall rate of 95.6% and a precision rate of 95.5%. Compared to models using a single data source, this multimodal model performs better. The study demonstrates that an effective intelligent method based on CNN can assist and standardize traditional manual interpretation, providing a reliable and innovative paradigm for cementing quality evaluation.

## 1. Introduction

Cementing operation is the process of effectively sealing the annular space between the casing string dropped in the well and the formation or outer casing using appropriate equipment, techniques, and cementing fluids. It is a very common operation performed in most oil wells during the drilling construction phase and serves as a key project linking drilling engineering and oil production engineering. The success or failure of cementing not only affects the success of the early drilling engineering of a well, but the quality of cementing also has a significant impact on the later production of oil and gas wells. If the quality of cementing is poor, it not only brings difficulties to subsequent drilling and oil testing, but also greatly affects the production lifespan of the oil well [1].

Variable Density Logging (VDL) images faintly display the full waveform of acoustic signals along the wellbore direction. Interpreters assess cement bonding conditions by identifying the energy strength and continuity of casing waves and formation waves in the images. However, traditional cementing quality evaluation methods have significant limitations: 1) High Subjectivity: The interpretation results heavily rely on the professional knowledge and accumulated experience of logging interpreters. For complex bonding conditions, conclusions drawn by different interpreters may vary significantly. 2) Low Efficiency: Manually analyzing logging data segment by segment over thousands of meters is time-consuming and labor-intensive, making it difficult to meet the demand for rapid evaluation of large numbers of oil and gas wells. 3) Insufficient Utilization of Information: The human eye struggles to capture all intuitive and subtle features in the data, which may lead to incomplete information utilization and misjudgments.

Recently, with the advancement of artificial intelligence technology, machine learning methods have been explored in the field of logging interpretation, such as Support Vector Machines (SVM) and Random Forests. Although these methods have achieved some success, they often require complex manual feature engineering—i.e., predefining features that can characterize cementing quality. This process itself is a challenge and limits the generalization ability of the models.

Deep learning, particularly Convolutional Neural Networks (CNNs), has made groundbreaking progress in areas such as image recognition and speech recognition. The core strength of CNNs lies in their ability to automatically learn and extract hierarchical features from raw data—from low-level to high-level—through multilayer convolution and pooling operations. This eliminates the need for cumbersome manual feature engineering. Since VDL data is essentially a two-dimensional image, it is highly suitable for processing with CNNs. To ensure the success of cementing operations, it is essential to test the quality of the cement sheath. So far, the only method to determine the bonding quality of the cementing sheath is pressure testing [2]. However, field experience has shown that pressure testing may cause damage to the cement sheath [3]. Currently, amplitude-variable density logging is the main method for evaluating cementing quality. However, the process of interpreting cementing quality through amplitude-variable density logging is complex and must be manually interpreted by trained experts, which also results in a large consumption of expert manpower for cementing interpretation [4]. At the same time, the next step of oil and gas well development will depend on the evaluation results of cementing quality, so cementing interpretation must be completed as soon as possible in a short time. Manual interpretation is slow and inefficient, and there is an urgent need for an efficient and accurate intelligent evaluation method for cementing quality [5].

In recent years, artificial intelligence algorithms have rapidly developed and have significant advantages in image recognition and big data analysis and processing. Many scholars at home and abroad have applied machine learning algorithms in the field of wellbore quality interpretation and have achieved good results [6–9] used neural networks to extract information from raw well logging data and rebuild raw acoustic well logging data. In the same year, Belozerov et al. 2025 used neural networks to identify reservoir locations from logging data. [10] used support vector machines and neural networks to automatically recognize ultrasonic waveform features, which can predict additional information about the longitudinal wave speed of annular materials in casing wells. [11] established different machine learning algorithms, such as random forests and neural networks based on amplitude, variable density logging data, and ultrasonic imaging data for prediction, outputting cementing quality prediction results. [12] used the Gaussian process regression algorithm for training, based on CBL and VDL logging data, generated new feature curves to accurately evaluate cementing quality. [13] proposed a cementing quality prediction method based on the GA-SVR algorithm, which can make advanced predictions of wellbore quality before cementing construction. [14] constructed an LSTM-BP serial neural network to calculate formation pore pressure in real-time. Fang et al. [15] established a convolutional neural network using only VDL images and achieved a commendable accuracy of 90%. While this demonstrated the potential of CNNs for this task, our work aims to further improve upon this by incorporating multimodal data. [16] came up with a Wasserstein distance generative adversarial network oriented to logging variable density images to expand the variable density image dataset.

Therefore, this paper aims to explore the use of deep learning technology to address the issue of cementing quality evaluation. We propose a multimodal intelligent detection model based on CNN, named CemQ-CNN. This model not only leverages CNN’s powerful image feature extraction capabilities to process VDL images but also extracts key numerical features from acoustic logging curves. The main contributions of this paper are as follows. Design and Implementation of an End-to-End Deep Learning Framework: A framework is developed for the automatic classification of cementing quality. Proposal of a Multimodal Input Strategy: This strategy integrates features from VDL images and acoustic logging curves, enhancing the model’s classification performance. Validation through Experiments: The effectiveness and rationality of the proposed method are verified through experiments conducted on a real labeled dataset containing 5,000 samples. Proposal of a Domain-Specific CNN Architecture: Unlike generic image recognition models, our CNN branch incorporates a multi-scale filter design that mimics the analytical process of human interpreters, who simultaneously evaluate both broad patterns and fine details in VDL images.

## 2. Evaluating the quality of cementing

After drilling, logging tools are lowered into the wellbore to collect logging data, which includes various physical parameters. This data is then processed and interpreted. This process requires logging analysis experts to combine geological knowledge and personal experience to convert logging data into geological information in order to accurately understand the geological conditions around the wellbore [17].

**Fig 1:**
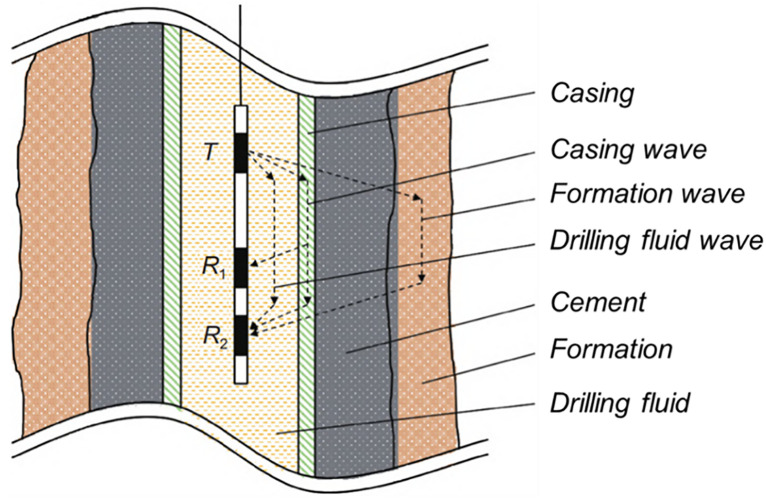
Principle of sound amplitude variable density logging.

Acoustic variable density logging is a type of acoustic logging [18]. Its principle is to reflect the cementing quality between the cement and casing, and between the casing and formation by utilizing the significant difference in acoustic impedance between cement and mud to affect the attenuation of sound waves propagating along the casing axis. The principle of amplitude-variable density logging is shown in Fig 1.

**Fig 2:**
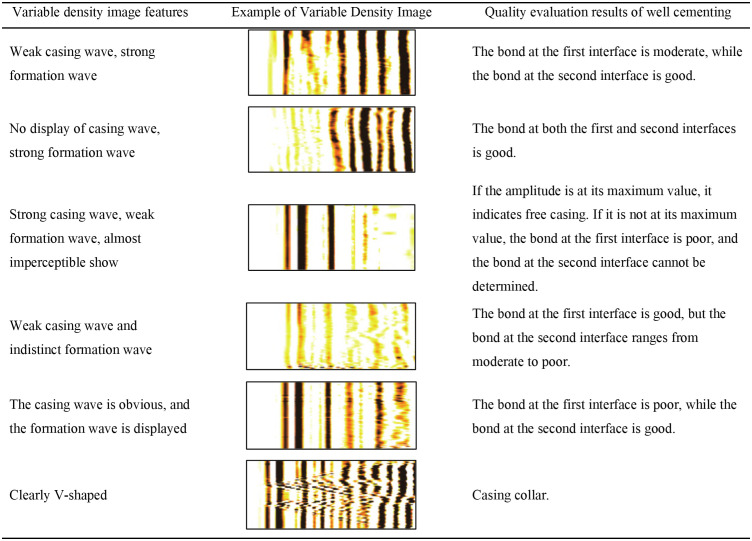
Example of cementing quality interpretation.

In acoustic amplitude logging (CBL), the source-to-receiver distance is 3 ft, with receiver R1 detecting casing waves. For variable density logging, the distance is 5 ft, and receiver R2 captures casing, cement sheath, formation, and direct waves. The cementing quality of the first bonding interface is assessed using CBL relative amplitude: below 20% indicates good bonding; 20%−40% suggests moderate quality; above 40% signifies poor bonding. The second interface is evaluated qualitatively. Stronger casing waves indicate poorer first interface bonding, while stronger, continuous formation waves suggest better second interface bonding. Variable density image characteristics are categorized into six types based on logging examples, as shown in Fig 2.

Variable density images possess a distinctive characteristic, featuring alternating light and dark bands that lack precise, objective values. The interpretation of the bands’ shape and brightness heavily relies on the subjective judgment of the evaluator. Consequently, the assessment outcomes are often influenced by personal biases, leading to inconsistent conclusions among different logging interpretation experts. To mitigate the subjectivity inherent in manual analysis, logging companies typically assign multiple interpreters to collaboratively evaluate the same logging data, aiming for more reliable results. However, this approach significantly increases the time and labor required for the process. As a result, achieving accurate and efficient cementing quality evaluation remains a formidable challenge. Developing an automated interpretation model for amplitude-variable density logging data offers a promising solution to enhance both the precision and efficiency of cementing quality assessment.

In acoustic logging curve, the cementing quality of the interface is evaluated using the relative amplitude method. The criteria are as follows: Relative Amplitude < 20%: Indicates good bonding. Relative Amplitude 20% – 40%: Suggests moderate bonding quality. Relative Amplitude > 40%: Signifies poor bonding.

The relative amplitude is a normalized measure that compares the acoustic signal amplitude measured at a specific depth in the well with a reference amplitude. This reference is the signal amplitude measured in a section of “free pipe”—a part of the casing that is known to be un-cemented. The relative amplitude is calculated using the following formula:


$$R_{Am}=\frac{A_{measured}}{A_{freepipe}}\times 100\%$$ (1)

Where *R*_Am_ is relative amplitude. *A*_measured_ is measured amplitude, which is the amplitude of the casing signal recorded by the CBL tool at the depth. *A*_freepipe_ is free pipe Amplitude, which is the maximum amplitude recorded in a section of casing that has no cement behind it. This value serves as the 100% baseline, representing the signal strength with zero attenuation from cement.

Referring to the response characteristics of the amplitude variable density logging map in Fig 2, a method for annotating the cementing quality training set was developed, as shown in Table 1. The training labels were re labeled for the dataset.

**Table 1:** Annotation method for cementing quality training set.

Acoustic logging curve features	Variable density image features	Cementing Quality
Relative Amplitude > 40%	Strong casing wave, weak formation wave	Poor
Relative Amplitude > 40%	The casing wave is strong and numerous, while the formation wave is weak and appears wavy	Poor
Relative Amplitude < 20%	Weak casing wave, strong formation wave	Good
Relative Amplitude < 20%	Weak casing wave and visible formation wave	Good
Relative Amplitude 20% – 40%	Strong casing waves and visible formation waves	Medium
Relative Amplitude 20% – 40%	Both casing waves and formation waves are weak	Medium

The task of evaluating cementing quality using amplitude-variable density logging curves closely resembles standard image classification problems. In this context, variable density images are segmented and fed into a convolutional neural network for training, while amplitude data is processed through a multilayer perceptron for further analysis. This combined approach facilitates the classification of cementing quality with improved consistency and objectivity.

## 3. Methodology

In this section, we concentrate on CemQ-CNN model to complete the classification task. As can be seen from Fig 2, the evaluation of cementing quality has multiple solutions. Relying solely on variable density images cannot accurately determine the cementing quality. Therefore, amplitude data needs to be added as a new feature. Convolutional neural networks (CNN) extract local features from signals by performing convolution operations between the filter kernel and the local part of the input signal [Warren S, et al. 1943]. The filter kernel remains consistent when processing the input signal, producing the corresponding output feature map [19–22]. Each output map generated by the filter represents a feature map of the next layer, and the depth of this layer is determined by the number of filter kernels. The mathematical formula for the convolution operation is shown in Eq. (2).


$${𝐅}_{l}=\sigma \left({𝐖}_{l}*{𝐅}_{l-1}+{𝐛}_{l}\right)$$ (2)

where **F**_l_ and **F**_l−1_ denote feature maps at layers *l* and *l*-1, σ represents a nonlinear activation function, **W**_*l*_ designates a kernel matrix, * denotes the convolution operation, and **b**_*l*_ is the bias. Feature maps at different layers are also functions of the input map **m** (A set of wells’ drainage strategies), and we define F_0_ = **m**.

**Fig 3:**
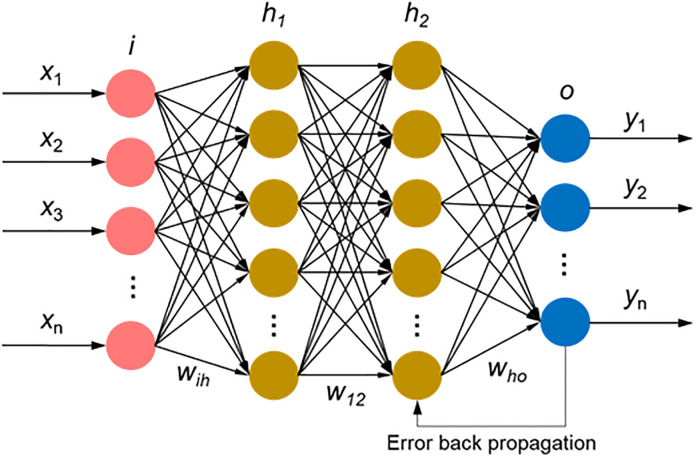
A multi-layer perceptron with four layers of neurons.

As shown in Fig 3, the layers of neurons are fully connected. Shelhamer, et al. 2017 proposed the error backpropagation neural network [23]. The multilayer feedforward network trained according to the error backpropagation algorithm is also one of its most widely used models.

**Fig 4:**
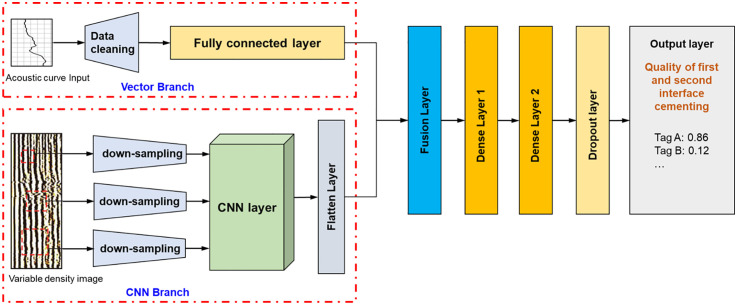
CemQ-CNN model architecture.

The CemQ-CNN model proposed in this paper is a multimodal fusion network, with its structure illustrated in Fig 4. It primarily consists of two parallel feature extraction branches and a fusion classification module.

VDL Image Processing Branch (CNN Branch): 1) This branch is designed to automatically extract spatial features from VDL images. It is composed of a series of convolutional layers (Conv), activation functions (ReLU), and max pooling layers (Max Pooling) stacked together. 2) Input Layer: Accepts grayscale images with dimensions of 256 × 256 × 1. Convolutional Layers: Utilizes small 3 × 3 convolution kernels to capture local details. The branch includes three convolutional blocks, each containing two convolutional layers followed by one max pooling layer. The number of filters increases progressively across the blocks (e.g., 32 → 64 → 128) to learn increasingly complex and abstract features. 3) Flatten Layer: Converts the multidimensional feature map output from the last convolutional block into a one-dimensional vector [23,24].

Acoustic Curve Feature Branch (Vector Branch): This branch is a simple fully connected network (or directly used as input) designed to process a 128-dimensional feature vector derived from acoustic logging curves.

Fusion & Classification Module: 1) Fusion Layer: Concatenates the feature vector obtained from the VDL image branch with the feature vector from the acoustic curve branch, forming a long vector that integrates information from both data modalities. 2) Fully Connected Layers: Following the fusion vector, two fully connected layers (Dense Layers) are connected, with ReLU as the activation function, to further integrate and learn features. To prevent overfitting, a Dropout layer (rate = 0.5) is inserted between the fully connected layers. 3) Output Layer: The final layer is a fully connected layer with 3 neurons, using the Softmax activation function to output the probabilities of the sample belonging to the three categories: “good,” “medium,” and “poor.”

To comprehensively evaluate the model’s performance, we adopted the following three widely used metrics:

Classification Accuracy (Accuracy) is shown in Eq. (3): The proportion of samples correctly classified by the model out of the total number of samples.


$$Accuracy=\frac{TP+TN}{TP+TN+FP+FN}$$ (3)

Precision is shown in Eq. (4): The proportion of samples predicted as positive by the model that are actually positive. For multi-class problems, we calculate the precision for each class and then compute its macro-average.


$$Precision=\frac{TP}{TP+FP}$$ (4)

Recall is shown in Eq. (5): The proportion of samples that are actually positive and are successfully predicted as positive by the model. Similarly, we compute its macro-average for multi-class problems.


$$Recall=\frac{TP}{TP+FN}$$ (5)

Where: TP — True Positive; FN — False Negative; FP — False Positive; TN — True Negative.

**Fig 5:**
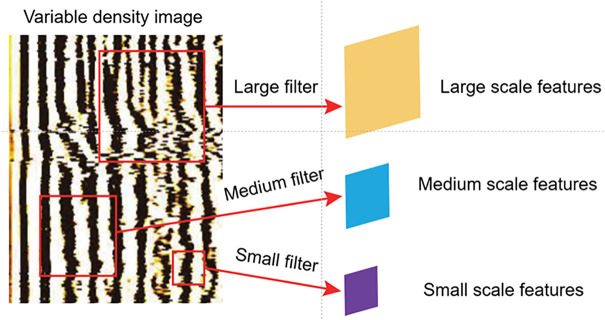
Multi-scale feature extraction using filters of different sizes.

At the same time, this article considers that logging variable density images are not simply image recognition tasks. When evaluating variable density, it is necessary to integrate the overall and local features of the image. That is, the evaluation results of well cementing quality are not only based on the brightness and darkness features of individual bands, but also refer to the shape and density of all bands. Therefore, this article improves the convolutional neural network part of the model, sets up filters of different sizes, extracts information of different scales in the image, as shown in Fig 5, and establishes a multi-dimensional feature extraction convolutional neural network, which can further improve the accuracy of the model. The settings for different filters are as follows:

1) Large filer: 128 filters, kernel size: 5 × 5, stride: 1 × 1, Activation function: ReLu
2) Medium filer: 128 filters, kernel size: 3 × 3, stride: 1 × 1, Activation function: ReLu
3) Small filer: 128 filters, kernel size: 1 × 1, stride: 1 × 1, Activation function: ReLu

Experimental Design for Validation: To systematically validate the contributions of our proposed multimodal and multi-scale architecture, we designed a set of ablation studies [25]. The performance of the full CemQ-CNN model is compared against two simplified baseline models: (1) VDL-only CNN model, which removes the acoustic curve branch to assess the impact of multimodal data, and (2) Vector-only MLP model, which uses only the acoustic curve features. This allows us to quantify the performance contribution of each data modality. The results are presented and discussed in Section 5.

Finally, we performed a 5-fold cross-validation. In each fold, 80% of the wells (~120 wells) were used for training, and the remaining 20% (~30 wells) were used for testing. The folds were stratified by geological block to maintain a representative distribution in each split. The full CemQ-CNN model was trained and evaluated five times, once for each fold.

## 4. Training procedure and evaluation

Dataset Construction: The data used in this study were collected from 150 wells across three distinct geological blocks within the oilfield, ensuring a diverse and representative dataset. As detailed in Table 2, these blocks encompass different formation types (sandstone, shale, carbonate) and varying well conditions (e.g., high pressure, high temperature), which present a range of cementing challenges. This diversity is crucial for training a robust model with strong generalization capabilities. A total of 5,000 representative cementing quality logging sections were curated. Each sample consists of a VDL image and its corresponding acoustic interpretation curve data. The distribution of “Good,” “Medium,” and “Poor” samples was kept relatively balanced across the different geological blocks to prevent the model from learning spurious correlations related to specific geological settings. The labeling process involved a two-stage expert-driven methodology. First, samples were provisionally categorized using quantitative industry standards from Cement Bond Log (CBL) amplitude data (e.g., amplitude < 20% suggesting ‘Good’). Second, a team of at least three senior logging interpreters reviewed each sample’s VDL image to make a final determination based on qualitative features (e.g., casing wave strength, formation wave continuity). When expert opinions differed, a consensus was reached through group discussion to ensure high-quality and consistent labels. The labels are categorized into three classes: “Good Cement,” “Medium Cement,” and “Poor Cement.” When expert opinions differed, a consensus was reached through group discussion to ensure label accuracy. Finally, the dataset includes 2,000 “Good” samples, 1,500 “Medium” samples, and 1,500 “Poor” samples.

**Table 2:** Summary of dataset provenance and geological characteristics.

Geological Block	Key Formation Type	Well Conditions	No. of Wells	No. of Samples (“Good”/”Medium”/”Poor”)
Block A	Sandstone	Normal Pressure, Shallow Depth	55	1850 (750/ 550/ 550)
Block B	Shale	High Pressure, High Temperature	60	2050 (800/ 650/ 600)
BlockC	Carbonate	High Pressure, High Temperature	35	1100 (450/ 300/ 350)

Data Preprocessing: 1) VDL Image Processing: To isolate the relevant data, a preprocessing step of cropping was performed. The raw VDL log images were manually cropped to select the core area that fully reflects the cementing interface, removing headers and depth tracks. All segmented images were then converted to single-channel (grayscale) and resized to a uniform resolution of 256 × 256 pixels. The input shape for our model is therefore (256, 256, 1), which is computationally more efficient than a standard 3-channel RGB input. Then, the pixel values were normalized by scaling them from [0, 255] to [0, 1] to accelerate model convergence. 2) Acoustic Curve Feature Extraction: For the acoustic amplitude and transit time curves corresponding to each VDL image section, we performed comprehensive feature engineering to capture their characteristics. For each of the two curves, we calculated a set of 64 statistical features. These included: basic statistical moments (mean, variance, skewness, kurtosis), range features (min, max, peak-to-peak amplitude), and percentile values (25th, 50th, 75th). To capture textural information, these statistics were computed over both the entire section and on smaller, overlapping sub-windows. The concatenation of these features from both curves resulted in a 128-dimensional feature vector. This dimension was empirically chosen to provide a rich description of the curve data without introducing excessive model complexity.

Dataset Splitting: All 5,000 samples were randomly divided into a training set (3,500 samples), a validation set (750 samples), and a test set (750 samples) with a ratio of 7:1.5:1.5. The network structure details are shown in Table 3. It should be noted that this random splitting tests the model’s ability to generalize to new, unseen logging sections from the same pool of wells. While a well-level split would be a stricter test of generalization to entirely new wells, our approach is valid for the common operational task of evaluating different zones within already drilled wells. The large number of wells (150) in our dataset helps mitigate the risk of the model overfitting to the specific characteristics of any single well.

**Table 3:** CemQ-CNN model architecture.

Layer Name	Layer Type	Output Shape	Parameter number	Connected to
**VDL Image Input**	InputLayer	(Batch size, 256, 1)	0	VDL Image Input
Conv1−1	Conv2D	(Batch size, 256, 256, 32)	320	VDL Image Input
Conv1–2	Conv2D	(Batch size, 256, 256, 32)	9248	Conv1–1/Relu
MaxPooling1	MaxPooling2D	(Batch size, 128, 128, 32)	0	Conv1–2/Relu
Conv2−1	Conv2D	(Batch size, 128, 128, 64)	18496	MaxPooling1/MaxPool
Conv2−2	Conv2D	(Batch size, 128, 128, 64)	36928	Conv2–1/Relu
MaxPooling2	MaxPooling2D	(Batch size, 64, 64, 64)	0	Conv2–2/Relu
Conv3−1	Conv2D	(Batch size, 64, 64, 128)	73856	MaxPooling2/MaxPool
Conv3−2	Conv2D	(Batch size, 64, 64, 128)	147584	Conv3–1/Relu
MaxPooling3	MaxPooling2D	(Batch size, 32, 32, 128)	0	Conv3–2/Relu
GlobalPool	GlobalAveragePooling2D	(Batch size, 128)	0	MaxPooling3/MaxPool
**Acoustic Curve Input**	InputLayer	(Batch size,128)	0	Acoustic_Curve_Input
Concatenate	Concatenate	(Batch size, 131200)	0	Flatten/Reshape:0, Acoustic Curve Input
Dense1	Dense	(Batch size, 512)	67174912	Concatenate/concat
Dropout	Dropout	(Batch size, 512)	0	Dense1/Relu
Dense2	Dense	(Batch size, 256)	131328	Dropout/Identity
Output	Dense	(Batch size, 3)	771	Dense2/Relu

Hyperparameter Search Space: We performed a grid search over a limited set of key hyperparameters. The ranges explored were: 1) Learning Rate (LR): [1e-4, 1e-3, 1e-2]; 2) Dropout Rate: [0.3, 0.5]; 3) Batch Size: [16, 32, 64]

Selection Protocol: The final hyperparameters (LR = 0.001, Dropout = 0.5, Batch Size = 32) were chosen based on the combination that yielded the lowest validation loss after 50 epochs during these preliminary experiments.

Reproducibility: We have now explicitly stated that all experiments were conducted using a fixed random seed (seed = 42) for TensorFlow, NumPy, and Python’s random module to ensure the reproducibility of our dataset splits and model weight initializations.

Early Stopping: The original manuscript mentioned our early stopping mechanism. We have re-emphasized that training was monitored on the validation set, with a patience of 10 epochs.

The model was implemented using the TensorFlow 2.0 framework and trained on an NVIDIA RTX 3090 GPU. We used the Adam optimizer with an initial learning rate of 0.001. The loss function was set to Categorical Cross-Entropy, which is commonly used for classification problems. The batch size was set to 32, and the training was conducted for 100 epochs. These core hyperparameters were selected based on preliminary experiments and established best practices, which indicated they provided stable convergence and strong performance for this task. Additionally, an early stopping mechanism was introduced. Training was terminated early if the loss on the validation set did not decrease for 10 consecutive epochs. This helped prevent overfitting and ensured the best model was saved.

## 5. Results and analysis

To comprehensively evaluate the performance of our proposed CemQ-CNN model, a series of experiments and analyses were conducted on the dedicated test set. The evaluation focuses not only on overall accuracy but also on class-wise performance, training dynamics, comparison with baseline models, and computational efficiency.

**Fig 6:**
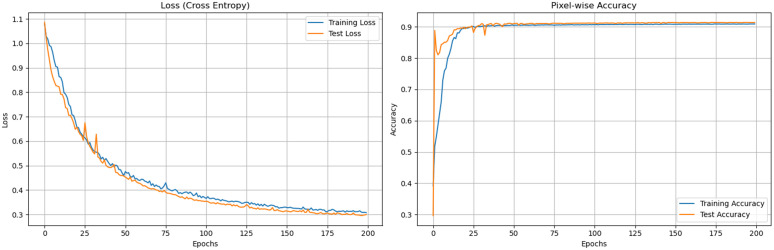
Training and validation loss and accuracy curves.

To assess the learning process and detect potential issues like overfitting or underfitting, we monitored the loss and accuracy metrics on both the training and validation sets throughout the 100 epochs. Fig 6 illustrates these training dynamics.

**Fig 7:**
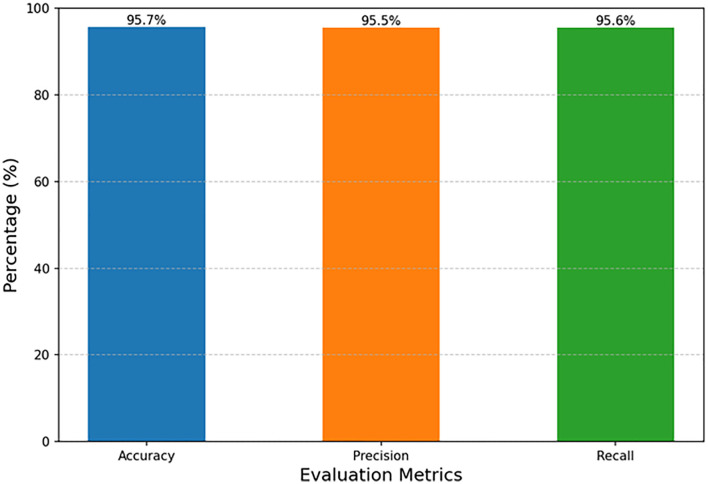
Overall performance of CemQ-CNN model on test set.

As depicted in Fig 7, both the training loss and validation loss consistently decreased, while their corresponding accuracies steadily increased over epochs. The curves show a smooth convergence, indicating that the model effectively learned from the training data. Crucially, the validation loss did not significantly diverge from the training loss, nor did the validation accuracy drop considerably, demonstrating the effectiveness of early stopping in preventing overfitting and ensuring robust generalization to unseen data.

We evaluated the trained CemQ-CNN model on the 750 test samples. The overall performance metrics—Accuracy, Macro Precision, and Macro Recall—are presented in Fig 4. Further detailed performance metrics, including precision, recall, and F1-score for each class (“Good,” “Medium,” “Poor”), are summarized in Table 3.

From Fig 7, it can be observed that the model achieved an overall accuracy of 95.7% on the test set, indicating strong generalization ability. The macro-average precision and recall both exceed 95%, demonstrating that the model maintains stable and Good performance across all categories, without significant bias toward any specific class.

Table 4 provides a more granular view of the model’s performance. The “Good” and “Poor” categories show exceptionally high F1-scores (0.972 and 0.964 respectively), indicating excellent balance between precision and recall for these distinct classes. The “Medium” class, while slightly lower, still maintains a strong F1-score of 0.932. This detailed breakdown confirms the model’s reliability in distinguishing between different cementing quality levels.

**Table 4:** Detailed classification report for CemQ-CNN model on test set.

Class	Precision	Recall	F1-Score	Support (Test Set)
Good	0.967	0.978	0.972	267
Medium	0.922	0.942	0.932	225
Poor	0.980	0.949	0.964	258
Macro Avg	0.955	0.955	0.955	750
Weighted Avg	0.957	0.957	0.957	750
Accuracy				0.957

**Fig 8:**
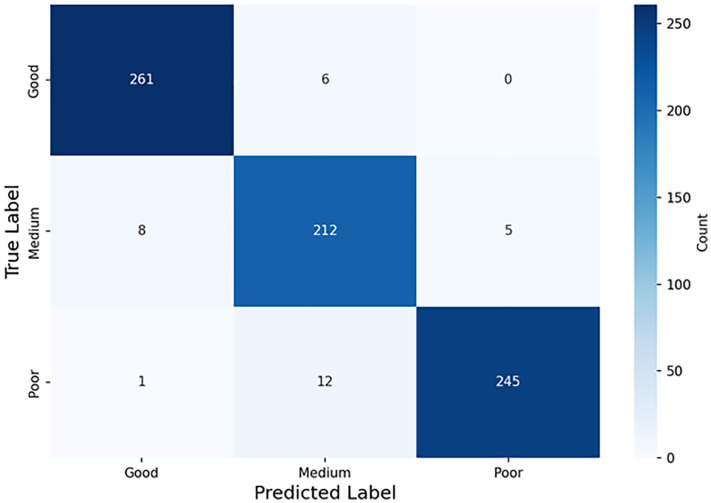
Confusion matrix on test set.

To gain deeper insights into the model’s specific performance across different categories and identify common misclassifications, we generated a confusion matrix for the test set, as shown in Fig 8 [26]. The total sample size of the test set is: Good (267), Medium (225), Poor (258).

From Fig 8, the values on the diagonal (in bold) represent the number of correctly classified samples, showing that these samples were all correctly classified. The model demonstrates the strongest recognition ability for the “Good” class, with a recognition rate as high as 97.8%. The primary confusion, as highlighted in the off-diagonal cells, occurs at the boundary of the “Medium” class. Specifically, 12 “Poor” samples were misclassified as “Medium,” and 8 “Medium” samples were misclassified as “Good.” This phenomenon aligns with real-world interpretation challenges and can be attributed to specific feature ambiguity.

In these ambiguous cases, the VDL images of “Medium” quality samples often exhibit transitional features: the casing wave may be present but weaker than in a typical “Poor” case, while the formation wave may be visible but discontinuous, unlike the strong, clear signal of a “Good” case. Similarly, the statistical features from their acoustic curves (e.g., mean amplitude, standard deviation) tend to occupy an overlapping numerical range between the “Good” and “Poor” classes. This lack of a distinct feature boundary makes the “Medium” category inherently more challenging for both automated models and human experts to classify with perfect accuracy. Conversely, the interference between “Good” and “Poor” is extremely rare (only 1 case), indicating that the model can effectively distinguish between these two extreme cases. Interference between “Good” and “Poor” is extremely rare (only 1 case), indicating that the model can effectively distinguish between these two extreme cases.

Ablation Study: To validate the effectiveness of our proposed multimodal input strategy, we designed ablation experiments to compare the performance of different models: 1) CNN with VDL Only: A CNN model using only VDL images as input. 2) MLP with Vector Only: An MLP model using only acoustic curve profiles as input. 3) CemQ-CNN: Our proposed multimodal model that integrates VDL images and elliptical features.

**Fig 9:**
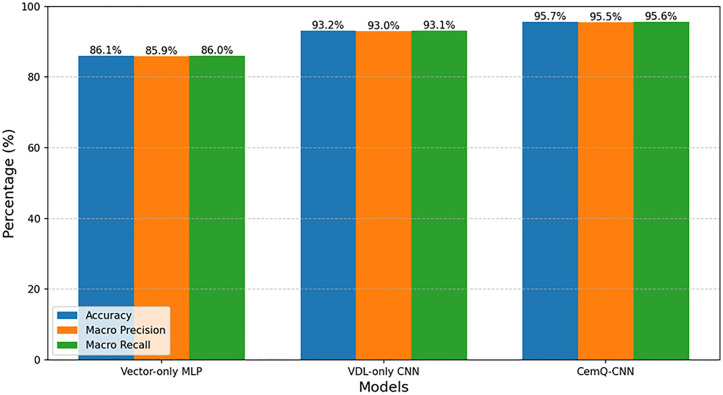
Performance comparison of different models (Ablation Study).

To validate the effectiveness of our proposed multimodal input strategy, we designed ablation experiments to compare the performance of different model architectures. The results, previously summarized in Fig 9, are further detailed in Table 4, showing Accuracy, Macro Precision, Macro Recall, and F1-score.

The MLP model, which relies solely on numerical features of acoustic curves, performed the worst, achieving an accuracy of 86.1%. This indicates that statistical values alone are insufficient to comprehensively reflect the complex nature of cementing quality.

The CNN model, which uses only VDL images, showed a significant performance improvement, with an accuracy of 93.2%. It is noteworthy that our VDL-only CNN model, with an accuracy of 93.2%, already surpasses the 90% accuracy reported by Fang et al. [15] on a similar task. This improvement can be attributed to our larger and more diverse dataset and a potentially more optimized network architecture. This confirms that VDL images are a critical source of information for evaluating cementing quality, and CNNs are effective in extracting their features.

The CemQ-CNN model proposed in this paper, which integrates both data sources, achieved the best performance, with an accuracy improvement of 2.5% compared to the VDL-only model. This demonstrates that the numerical features of acoustic curves provide valuable supplementary information to the model, and the multimodal fusion strategy is both effective and necessary.

The experimental results in Table 5 clearly demonstrate the following: The MLP model, which relies solely on numerical features of acoustic curves, performed the worst, achieving an accuracy of 86.1%. This indicates that statistical values alone are insufficient to comprehensively reflect the complex nature of cementing quality. The CNN model, which uses only VDL images, showed a significant performance improvement, with an accuracy of 93.2%. This confirms that VDL images are a critical source of information for evaluating cementing quality, and CNNs are effective in extracting their features. The CemQ-CNN model proposed in this work, which integrates both data sources, achieved the best performance, with an accuracy improvement of 2.5% compared to the VDL-only model. This demonstrates that the numerical features of acoustic curves provide valuable supplementary information to the model, and the multimodal fusion strategy is both effective and necessary.

**Table 5:** Performance comparison of different models (Ablation study).

Model	Accuracy (%)	Macro Precision (%)	Macro Recall (%)	Macro F1-Score (%)
Vector-only MLP	86.1	85.9	86.0	86.0
VDL-only CNN	93.2	93.0	93.1	93.1
**CemQ-CNN**	**95.7**	**95.5**	**95.6**	**95.6**

To further contextualize the performance of CemQ-CNN, we benchmarked it against two widely used traditional machine learning algorithms in geological data interpretation: Support Vector Machines (SVM) and Random Forests (RF). For a fair comparison, both baseline models were trained on the same comprehensive feature set used by our multimodal model. This involved first extracting deep features from the VDL images using the pre-trained CNN branch of our CemQ-CNN model and then concatenating them with the 128-dimensional acoustic curve feature vector. This combined feature vector was then used as input for the SVM and RF classifiers. The results are presented in Table 6.

**Table 6:** Performance comparison with baseline machine learning models.

Model	Accuracy (%)	Macro Precision (%)	Macro Recall (%)	Macro F1-Score (%)
Support Vector Machine (SVM)	89.5	89.2	89.4	89.3
Random Forest (RF)	91.8	91.3	91.8	91.7
**CemQ-CNN**	**95.7**	**95.5**	**95.6**	**95.6**

As shown in Table 6, the CemQ-CNN model significantly outperforms the traditional machine learning models. The Random Forest model, with an accuracy of 91.8%, performs better than the SVM but still lags behind our proposed deep learning approach. This performance gap highlights the key advantage of an end-to-end deep learning framework [27]. While SVM and RF operate on a pre-defined feature set, CemQ-CNN can automatically learn and optimize hierarchical features directly from the raw VDL images and numerical data simultaneously. This integrated learning process allows it to capture more intricate and subtle patterns within the multimodal data, leading to a more accurate and robust cementing quality evaluation.

For practical deployment, computational efficiency is critical. The CemQ-CNN model was trained on an NVIDIA RTX 3090 GPU, with the full training process for 5,000 samples taking approximately 3.5 hours. While the training cost is non-trivial, the inference is highly efficient. Once trained, the model can classify a single VDL image and its corresponding acoustic vector in approximately 50 milliseconds. This speed enables near-real-time analysis, offering a significant efficiency advantage over manual interpretation which can take several minutes per section.

The ablation study in Table 6 confirmed that VDL images are a powerful data source for this task. To further validate the specific contribution of our proposed multi-scale CNN architecture, and to provide a more rigorous comparison against existing methodologies like that of Fang et al. [15], we conducted a controlled experiment.

We designed and implemented a baseline “Standard CNN” model. This model shares the same overall depth and number of parameters as our VDL-only branch but replaces our domain-specific, multi-scale convolutional layers with conventional single-scale (3x3) filters. This is a common and robust architecture for general image classification tasks. To ensure a fair comparison, this baseline model was trained and evaluated on the exact same training, validation, and test data splits as our proposed model. This head-to-head comparison effectively isolates the performance impact of the architectural design from variables like dataset size or labeling quality [28].

**Fig 10:**
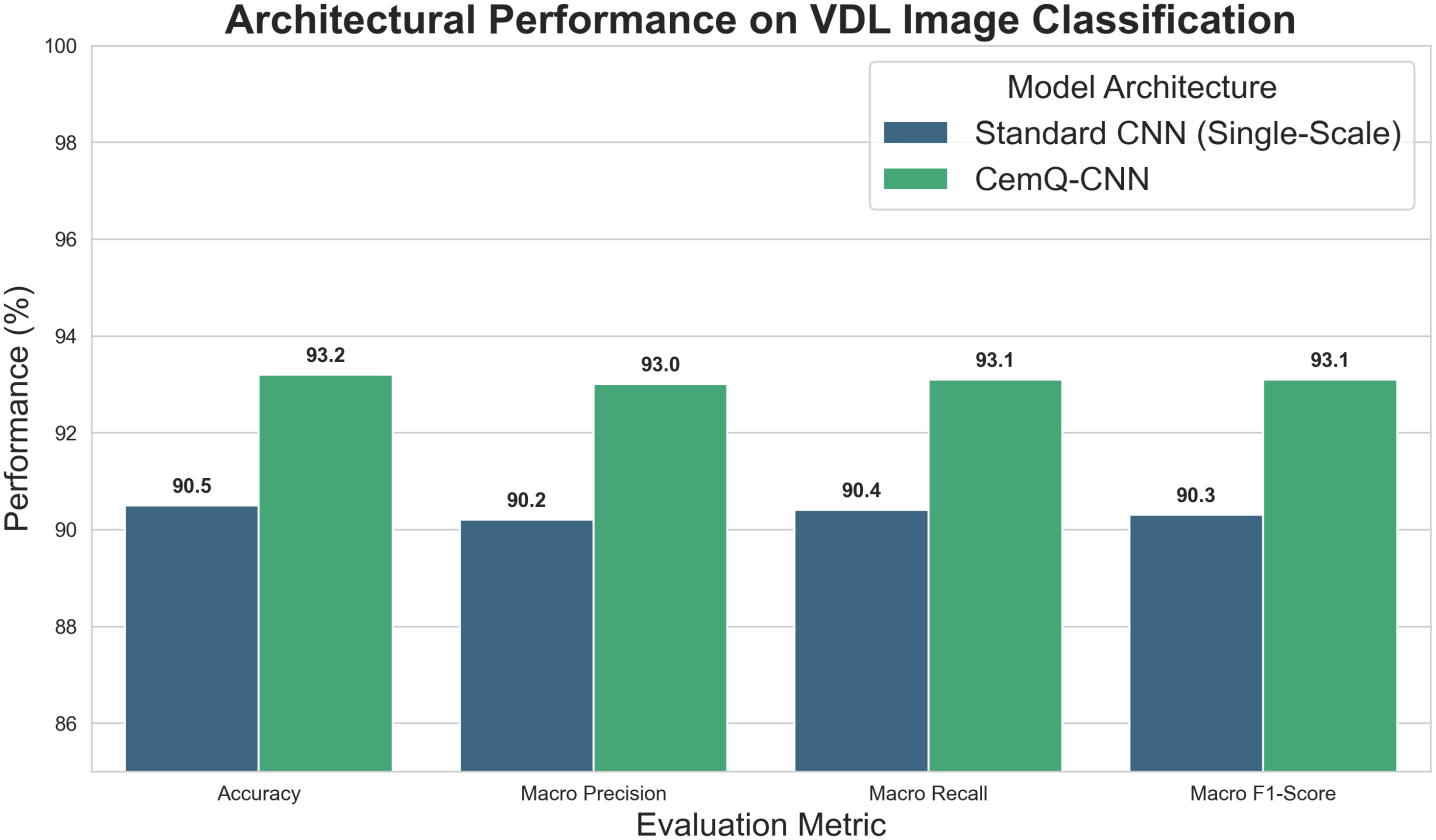
Architectural performance on VDL image classification.

The performance of both architectures on the VDL-only classification task is summarized in visualized in Fig 10.

The results clearly demonstrate the superiority of our multi-scale approach. The Proposed CNN achieved an accuracy of 93.2%, a 2.7% improvement over the 90.5% accuracy of the Standard CNN. Similar gains are observed across precision, recall, and F1-score.

This performance lift can be attributed to the multi-scale architecture’s ability to process VDL images in a manner that mimics human expert analysis. The larger filters (e.g., 5x5) capture the overall texture and continuity of formation waves (broad patterns), while the smaller filters (e.g., 1x1) focus on the sharpness and strength of individual casing wave bands (fine details). The Standard CNN, limited to a single scale, is less effective at simultaneously capturing this hierarchical information. This experiment empirically confirms that our domain-specific architectural choice is a key contributor to the model’s high accuracy and provides a more robust foundation for its comparison with prior work.

As shown in Table 7, there is a performance drop compared to the random split (95.7% vs. 91.5% accuracy). This drop is anticipated, as generalizing to new wells with potentially unique geological signatures or logging conditions is inherently more difficult. However, an accuracy of 91.5% is still a very strong result, demonstrating that our model possesses robust generalization capabilities and is not merely memorizing well-specific features. The low standard deviation across folds indicates stable performance.

**Table 7:** Well-Level 5-fold cross-validation performance.

Model	Mean Performance (%)	Standard Deviation (%)
Accuracy	91.5	± 1.8
Macro Precision	91.2	± 2.1
Macro Recall	91.3	± 1.9
Macro F1-Score	91.2	± 2.0

## 6. Conclusion

(1) This work addresses the issues of subjectivity and low efficiency in traditional cementing quality evaluation methods by proposing an intelligent detection approach based on a neural network model (CemQ-CNN). This method constructs a multimodal deep learning model that effectively integrates two core data sources—VDL images and acoustic curves—to achieve automatic and precise classification of cementing quality into three levels: “good,” “moderate,” and “poor.”
(2) Through experiments conducted on a real annotated dataset containing 5,000 samples, our model achieved a classification accuracy of 95.7% on the test set, along with good precision and recall rates. Ablation studies further confirmed that the multimodal strategy, which fuses features from VDL images and acoustic curves, significantly outperforms models relying on any single data source. These results fully demonstrate the effectiveness and reliability of the proposed method.
(3) The significance of this study lies in providing an objective, efficient, and intelligent solution for cementing quality evaluation. This approach can serve as a powerful tool to assist logging interpreters by greatly reducing their workload, improving interpretation consistency, and minimizing errors caused by fatigue or individual bias. It is important to acknowledge, however, that the model’s performance is contingent on the quality of the training labels. Since the labels were derived from expert consensus, the model inherently learns the patterns recognized by these experts, including any potential systematic biases. Therefore, the primary role of this tool is to ensure consistent application of established expert criteria at scale, rather than to eliminate subjectivity entirely.
(4) Future work can be explored in the following directions. Dataset Expansion: Incorporate logging data from diverse regions and well conditions to further improve the model’s generalization ability. Model Interpretability: Employ visualization techniques such as Grad-CAM to analyze the basis of the model’s decision-making process, thereby enhancing its credibility. Integration of Additional Logging Information: Explore the fusion of advanced logging data, such as perforation logging results, to build a more comprehensive evaluation model. To more rigorously test the model’s generalization capabilities to entirely new geological environments, future work should involve validation using a strict well-level or even field-level data split.

## Supporting information

S1 File Dataset links. (TXT)
